# Genome editing with the CRISPR‐Cas system: an art, ethics and global regulatory perspective

**DOI:** 10.1111/pbi.13383

**Published:** 2020-04-30

**Authors:** Debin Zhang, Amjad Hussain, Hakim Manghwar, Kabin Xie, Shengsong Xie, Shuhong Zhao, Robert M. Larkin, Ping Qing, Shuangxia Jin, Fang Ding

**Affiliations:** ^1^ National Key Laboratory of Crop Genetic Improvement Huazhong Agricultural University Wuhan China; ^2^ College of Public Administration Huazhong Agricultural University Wuhan China; ^3^ Key Laboratory of Agricultural Animal Genetics, Breeding and Reproduction Ministry of Education Wuhan China; ^4^ Key Laboratory of Horticultural Plant Biology Ministry of Education College of Horticulture and Forestry Sciences Huazhong Agricultural University Wuhan China; ^5^ Hubei Key Laboratory of Plant Pathology College of Plant Sciences and Technology Huazhong Agricultural University Wuhan China

**Keywords:** genome editing, CRISPR‐Cas system, ethics, regulations, risks

## Abstract

Over the last three decades, the development of new genome editing techniques, such as ODM, TALENs, ZFNs and the CRISPR‐Cas system, has led to significant progress in the field of plant and animal breeding. The CRISPR‐Cas system is the most versatile genome editing tool discovered in the history of molecular biology because it can be used to alter diverse genomes (e.g. genomes from both plants and animals) including human genomes with unprecedented ease, accuracy and high efficiency. The recent development and scope of CRISPR‐Cas system have raised new regulatory challenges around the world due to moral, ethical, safety and technical concerns associated with its applications in pre‐clinical and clinical research, biomedicine and agriculture. Here, we review the art, applications and potential risks of CRISPR‐Cas system in genome editing. We also highlight the patent and ethical issues of this technology along with regulatory frameworks established by various nations to regulate CRISPR‐Cas‐modified organisms/products.

## The art of CRISPR‐Cas system

**Figure 1:**
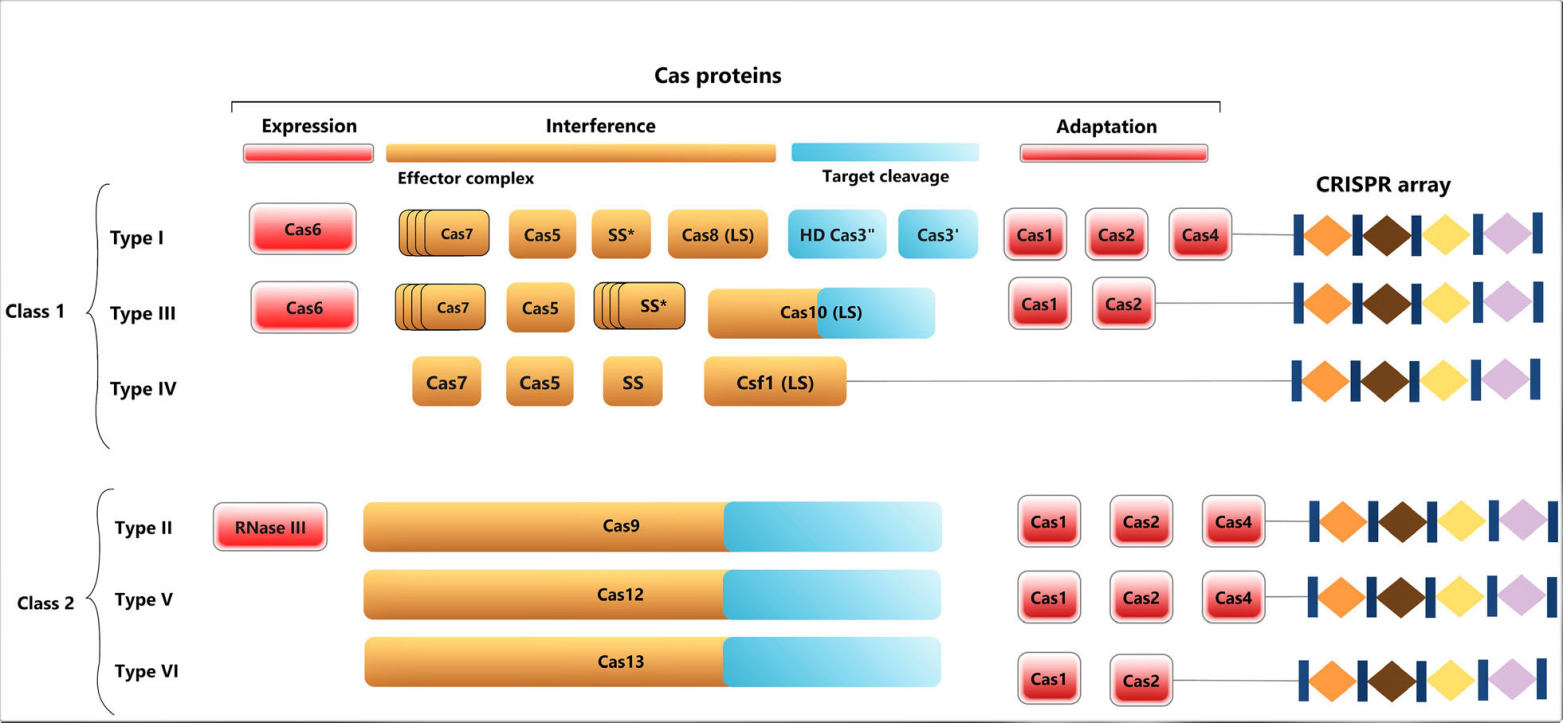
Latest classification of CRISPR‐Cas systems. Generally, CRISPR‐Cas system is comprised of two parts: a set of Cas proteins, involved in immunity, and the CRISPR array, which consists of direct repeats and spacers. The CRISPR array helps to remember the invader. On the basis of Cas proteins, CRISPR‐Cas system is divided into two classes, class 1 and class 2. Class 1 CRISPR‐Cas systems consist of a set of effector complexes, whereas class 2 systems contain a single protein. Each class includes three subtypes—type I, III and IV in class 1 system and type II, V and VI in class 2 system.

Genome editing has revolutionized DNA manipulation in eukaryotes enabling the precise mutagenesis of single base pairs, the introduction of insertions and/or deletions (indels), DNA fragment substitution and the nucleotide base conversion (Carroll, 2017; Naso and Petrova, 2019). The most frequent genome editing systems involve ODM (oligonucleotide‐directed mutagenesis; Sauer *et al.*, 2016), TALENs (transcription activator‐like effector nucleases), ZFNs (zinc finger nucleases) and CRISPR‐Cas (clustered regularly interspaced short palindromic repeats and CRISPR‐associated proteins; Carroll, 2014; Petolino, 2015). TALENs and ZFNs are still in use for research in various agricultural and medical arenas. However, the applications of these technologies are limited because of the transfection inefficiency, design complexity and limitations on multiplexed mutations (Doudna and Charpentier, 2014). In contrast, the most powerful and game‐changing technique, the CRISPR‐Cas system, is currently the dominant genome editing technology in research laboratories around the world due to its efficiency, precision and broad application range (Ledford, 2015). The CRISPR‐Cas system was discovered as a form of adaptive immunity system in bacteria and archaea to defend against invading viral, plasmid and/or phage DNA (Albitar *et al.*, 2018; Ishino *et al.*, 1987; Sovová *et al.*, 2016). CRISPR‐Cas system is typically comprised of two parts: (i) Cas proteins—involved in protection and the acquisition of invading nucleotides, and (ii) the CRISPR array—which consists of conserved recurrent domains known as direct repeats and imbedded variable sequences with same length known as spacers (Figure 1). The CRISPR array helps to remember the invader (Wang *et al.*, 2019b). The CRISPR‐Cas system usually works in a specified‐sequence way by recognizing and cleaving foreign nucleotides. As a defence mechanism, CRISPR‐Cas has three steps: (i) adaptation: invading nucleotide fragments are captured by the host organism; subsequently, associated insertions are designed within the CRISPR array, (ii) expression/crRNA biogenesis: CRISPR array is transcribed into a large precursor crRNA (pre‐crRNA) that is cleaved by RNases to produce a mature guide crRNA and (iii) interference: the mature crRNA is used to guide effector complexes that capture and degrade invading nucleotides (Hille and Charpentier, 2016; Wang *et al.*, 2019b).

The CRISPR‐Cas system has been classified into 2 classes based on their effector proteins (Figure 1). Class 1 systems consist of a set of effector complexes with 4 to 7 Cas proteins, while class 2 systems are comprised of only one Cas protein involving multiple sub‐domains. Each of these classes includes three subtypes—type I, III and IV in the class 1 system, while type II, V and VI in the class 2 system (Ishino *et al.*, 2018). Type I, II and V target DNA and type VI targets RNA (Gasiunas *et al.*, 2012; Koonin *et al.*, 2017). Type III recognizes and cleaves both DNA and RNA (Samai *et al.*, 2015). Due to simple structural design of effector complexes, class 2 CRISPR‐Cas systems have become an attractive choice to develop an innovative generation of genome editing technology.

**Figure 2:**
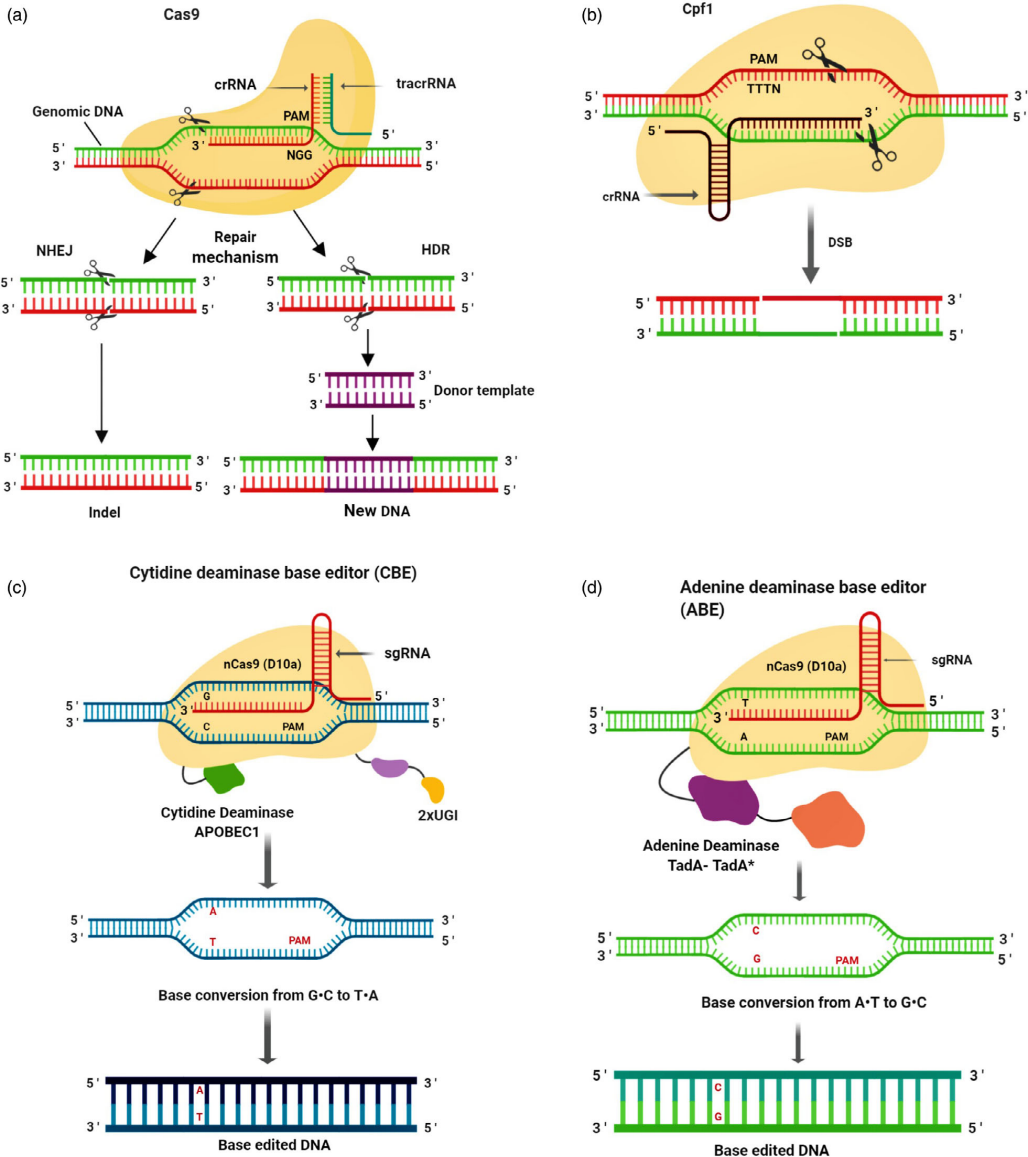
Diagrammatic representation of CRISPR‐Cas9 system. (a) CRISPR‐Cas9 is a sgRNA‐dependent endonuclease. In the presence of sgRNA, Cas9 binds to the target site, close to a short DNA sequence motif called PAM, and creates a double‐stranded break. In a result, the cell initiates two mechanisms to repair the break: NHEJ, produces indels and loss‐of‐function mutations and the other mechanism, HDR which corrects a pre‐existing mutation by introducing a template DNA sequence that helps to repair the break by copying the template sequence. (b) In Cpf1 system, a ‘TTTN’ rich region is required which acts as PAM. It creates double‐stranded breaks with staggered ends. (c) Cytidine deaminase base editing (CBE) system linked with APOBEC1 cytidine deaminase converts G•C base pair to T•A base pair at targeted loci. (d) Adenine deaminase base editing (ABE) system linked with adenine deaminase converts A•T base pair to G•C at specific target loci.

The best studied and most frequent multiple‐domain protein is Cas9, a crRNA‐dependent endonuclease—containing two distinct nuclease domains, that is HNH and RuvC. These nuclease domains cleave target and non‐target DNA strands, respectively (Ishino *et al.*, 2018). CRISPR‐Cas9 is able to introduce double‐stranded breaks (DSB) at target sites in the genomic DNA. Cas9 is followed by guide RNA (gRNA) to a specific DNA sequence where it cuts both strands (Braff *et al.*, 2016). If target sequence is homologous to gRNA, Cas9 will bind at the chosen genomic locus adjacent to a short DNA sequence known as PAM (protospacer adjacent motif) and create a DSB (Chneiweiss *et al.*, 2017). The cell typically uses two mechanisms to repair the DSB. One of these mechanisms is non‐homologous end joining (NHEJ), which usually generates indels that result in loss‐of‐function mutations. The other mechanism is homology‐directed repair (HDR), which corrects a pre‐existing mutation by introducing a template DNA sequence that helps to repair the break by inserting the template sequence (Figure 2a; Roy *et al.*, 2018).

In addition, a recently introduced class II type V endonuclease Cpf1 (CRISPR from *Prevotella* and *Francisella* 1) system has unique biochemical features that serves as an attractive tool for genome editing (Figure 2b). Cpf1 uses 5′‐TTTN‐3′ PAM sequence that increases the repertoire of protospacers (Mahfouz, 2017; Tang *et al.*, 2017). This system generates five nucleotide 5′‐OH cohesive overhangs in the distal end of the target sequence relative to the PAM (Vanegas *et al.*, 2019). These staggered DNA ends can enhance the efficiency of addition and replacement of a chosen DNA sequence at the cleaved site via HDR mechanism. It increases gene insertion at precise locus in the genome, offering a greatly desirable option that is however presently challenging in plants (Zaidi *et al.*, 2017). The protospacer of Cpf1 is longer than Cas9 and may therefore be more specific (Kleinstiver *et al.*, 2016). Cpf1 is a simple genome editing tool because the crRNA of this system consists of a protospacer which functions directly as a gRNA (Kleinstiver *et al.*, 2016; Vanegas *et al.*, 2019).

The current genome editing systems have significantly contributed to an efficient and precise editing of desired DNA sequence with their efficiency to generate DSBs, but the mechanism of DNA repair via NHEJ may result in random indels or other potential unwanted DNA rearrangements at DSB sites (Kosicki *et al.*, 2018). More recently, novel chimeric CRISPR/Cas9‐derived programmable base editing platforms have been designed for precise nucleotide conversion (Naso and Petrova, 2019). These systems involve different catalytic activity, architecture and potential modifications. These have offered new possibilities for engineering single base changes, creating novel protein variants, diversifying a localized sequence and accelerating the evolution of specific proteins (Hess *et al.*, 2016; Zong *et al.*, 2017). These efforts can expand the development of traits and enhance the trait range in agriculture as well as in humans for the design of gene therapies for several genetic disorders.

Comparing with DSB‐HDR‐mediated genome editing, base editing systems comprise site‐directed alteration of DNA base pairs along with manipulating a DNA repair mechanism to achieve a precise repairing of edited base (Hess *et al.*, 2017; Manghwar *et al.*, 2019). Base editors are chimeric proteins that consist of a catalytic domain and a DNA targeting unit which deaminate the adenine or cytidine base. These systems do not require DSBs to modify DNA base pairs and, therefore, limit the creation of indels at target and off‐target sites (Komor *et al.*, 2017). In most of these systems, the DNA targeting unit involves dCas9 guided by a sgRNA (Hess *et al.*, 2017). Cas9 nickase is also used as a targeting unit which allows for higher frequency of base editing (Eid *et al.*, 2018; Ran *et al.*, 2013).

In this review, we consider two DNA editing systems: the cytidine deaminase‐based editing (CBE) and the adenine deaminase‐based editing (ABE) systems. Cytidine deaminases were developed to convert cytosine into uracil (Shimatani *et al.*, 2017). In this system, APOBEC (apolipoprotein B mRNA editing enzyme), directed by dCas9, deaminates a specific cytidine to uracil and the subsequent U‐G mismatches are corrected through repair processes to form U‐A base pairs that results in T‐A base pairs (Figure 2c). Thus, these base editors can be utilized to generate C to T point mutations (Eid *et al.*, 2018; Qin *et al.*, 2019). Currently at the fourth generation of base editors, Cas9‐CBE4 involves a catalytically inactive Cas9 (dCas9) joined to an APOBEC cytidine deaminase which converts C‐G bases to T‐A bases in a 5‐bp window between the fourth and eighth base pair of the nonbinding strand of the sgRNA. The latest generation of CBE base editors has higher accuracy and greater efficiency than previous versions due to the inclusion of an inhibitor that prevents uracil‐N‐glycosylase (UNG) from altering DNA inadvertently (Naso and Petrova, 2019).

Complementary to CBEs, ABEs were designed for the modification of adenine bases (Gaudelli *et al.*, 2017). The initial version of ABEs was generated by an antibiotic resistance complementation method in bacteria, wherein the adenine editing domain of edTAd‐cas9 was mutated to insert the targeted adenine into a transformed chloramphenicol resistance gene. Thus, the manipulation of antibiotic selective pressure resulted in the creation of ABEs comprising different editing windows of activity (Eid *et al.*, 2018). Broad directed evolution and protein engineering led to the seventh generation of ABEs (ABE7.10) that mediates targeted A‐T to G‐C conversion within a specific activity window (Gaudelli *et al.*, 2017; Hua *et al.*, 2020). The tRNA adenine deaminase (TadA) from *E. coli* has been used to generate ABEs, which catalyses adenine deamination on ssDNA and converts adenine into inosine (Shi *et al.*, 2017). The deamination of adenosine produces inosine, which is joined to cytidine and subsequently corrected to guanine, which as a result, converts A to G, or A‐T to G‐C (Figure 2d; Eid *et al.*, 2018).

The CRISPR‐Cas system is highly versatile genome editing tool with the ability to modify a broad range of genomes with greater efficiency and accuracy, but due to recent pace and scope of research using the CRISPR‐Cas9 and its possible clinical applications, it is considered a ‘*disruptor’* technology (Ledford, 2015). The supercharged scientific and ethical commentaries highlight the intensity of the disagreements over the potential applications of CRISPR‐Cas9, especially when using it to modify human embryos for developmental research (Callaway, 2016) and germ‐line gene therapy (Liang *et al.*, 2015). Additionally, CRISPR‐Cas applications have raised moral and ethical issues in agriculture, livestock, industrial biotechnology, biomedicine and reproduction (Mulvihill *et al.*, 2017; Nuffield Council on Bioethics, 2016). In this review, we describe applications of the CRISPR‐Cas system in agriculture, animal science and plant biology. In addition, we have highlight the potential risks, patent issues and ethical considerations of the technique, along with regulatory policies developed in different countries for applications of CRISPR‐Cas system in genome editing.

## Potential risks of CRISPR‐Cas system

Despite the CRSPR**‐**Cas system is a highly efficient genome editing tool both for the genetic improvement of plants and animals and for the clinical investigation of human genetic diseases, it also poses potential risks in certain cases. In this context, we have highlighted two key concerns for this technology: off‐target editing and illegal or irresponsible experimentation by the scientific community. Regarding the first issue, several studies have demonstrated off‐target effects of CRISPR‐Cas9‐based genome editing in biomedical and clinical applications (Ma *et al.*, 2016; Saha *et al.*, 2018; Zhang *et al.*, 2015), and in functional studies of plant genome (Anderson *et al.*, 2016; Li *et al.*, 2018). Several reports have shown off‐target effects of CRISPR‐Cas9 in human, mouse and rat cell lines (Anderson *et al.*, 2018; Aryal *et al.*, 2018; Fu *et al.*, 2013). Recent studies on other CRISPR‐Cas systems, such as RNA‐guided base editing systems, have also indicated off‐target activities in mammals (Grünewald *et al.*, 2019; Kim *et al.*, 2019; Zuo *et al.*, 2019). Off‐target editing presents serious issues in therapeutics because it may causes loss‐of‐function mutations in proper functional genes or incorrect‐repairing of disease‐inducing gene due to binding and breaking at sites other than the target DNA sequence (Cox *et al.*, 2015). Off‐target editing may also lead to chromosomal rearrangements and other types of mutations, including the integration of DNA mismatches into the PAM‐distal position of the sgRNA sequence (Cong *et al.*, 2013; Ghosh *et al.*, 2019).

Nevertheless, the current status of CRISPR‐Cas9 applications in plant genome editing provides evidence that the frequency of off‐target mutation is quite low (Eş *et al.*, 2019). A recent study on *Arabidopsis thaliana* revealed unexpected off‐target editing by CRISPR‐Cas9 (Zhang *et al.*, 2018). While the onset of off‐target mutations is a major risk posed by CRISPR‐Cas system, researchers are currently paying attention to reduce off‐target editing (Schulman *et al.*, 2019). Recent studies of Feng *et al. *(2018) and Lee *et al. *(2019) found that off‐target mutations by CRISPR‐Cas systems are very rare or undetectable. Transgenic soybean developed through Agrobacterium‐mediated transformation revealed that off‐target editing generated less genetic variation than radiation mutagenesis (Anderson *et al.*, 2016). In addition, a recent study conducted by Li *et al.* (2019c) showed very few off‐target modifications in cotton plants edited by CRISPR‐Cas9. More specifically, this study revealed only four off‐target indel mutations among the 4413 potential off‐target sites that were validated by Sanger sequencing. These studies indicate that off‐target effects of the CRISPR‐Cas system in plants are very rare, with a few low‐frequency off‐target mutations. However, great care is needed concerning the detection of off‐target effects prior to any CRISPR‐Cas application particularly in human genome editing (Manghwar *et al.*, 2020).

A second concern with the CRISPR‐Cas system is the possibility of illegal or irresponsible experimentation and misconduct of CRISPR‐Cas technology. A Chinese scientist, Jiankui He, claimed in November of 2018 at the 2^nd^ International Summit on Human Genome Editing in Hong Kong, that he used CRISPR‐Cas9 to create genetically modified babies immune to the human immunodeficiency virus (HIV) (Ye *et al.*, 2019).

He’s experimentation on humans was criticized by various national and international academies and researchers. The Scientific Ethics Committee of the Academic Division of the Chinese Academy of Sciences posted a statement declaring their opposition to any clinical use of genome editing on human embryos, noting that ‘the theory is not reliable, the technology is deficient, the risks are uncontrollable, and ethics and regulations prohibit the action’ (Li *et al.,* 2019d). Moreover, the Chinese Academy of Medical Sciences stated a clear opposition to clinical applications of embryo genome editing, stating that: ‘it is opposed to any clinical operation of human embryo genome editing for reproductive purposes in violation of laws, regulations, and ethical norms in the absence of full scientific evaluation’ (Ma *et al.*, 2019; Wang *et al.*, 2019a).

He’s work breached Chinese regulations and violated other ethical and regulatory norms. China’s guidelines and regulations have already prohibited germ‐line genome editing to produce human embryos for clinical use due to scientific and ethical concerns (Li *et al.,* 2019d). Until the outcomes of genome editing can be better controlled, the CRISPR‐Cas9 system cannot be used for germ‐line gene modifications in humans. Although much is known about the CRISPR‐Cas9 technology, current knowledge about its targeting efficacy is still inadequate. Indeed, the explicit prohibition of using CRISPR‐Cas9 for human germ‐line genome editing in a clinical setting is primarily based on safety and efficacy concerns (Li *et al.,* 2019b). Additionally, off‐target mutations induced by CRISPR‐Cas9 may influence the edited organism and potentially the organism's descendants (Wang *et al.*, 2015). Moreover, future research and advances on this technique may reveal new unexpected off‐target effects that could lead to unpredictable consequences in future. These off‐target modifications can possibly result in cancer or other severe complications in early or later lives of edited babies (Kim *et al.*, 2015; Ma *et al.*, 2019). (Lanphier *et al.*, 2015). Therefore, the scientific community must respect the dignity of human life and remain sensitive to the dangers that research might cause, particularly to participants and also to the broader community. Biomedical researchers and other practitioners should follow the relevant laws and regulations. They should firmly hold to the ethical guidelines established for the safe translation of scientific research to human health (Li *et al.,* 2019d).

## Applications of the CRISPR‐Cas system

The first generation of genome editing tools that provided the recognition of target sequences was the ZFNs and TALENs, which appeared some 20 years ago. The CRISPR‐Cas system has outperformed the ZFN and TALEN systems in its capacity to modify genomic DNA (Waryah *et al.*, 2018) and thus has revolutionized the genome editing field (Jinek *et al.*, 2012). Plant, animal and even human genomes have been altered using the TALEN, ZFN and CRISPR‐Cas systems. These systems are now commonly used to edit particular sequences of genes and have impacted several fields. These systems have been used to alter the structure and regulation of genes, to perform gene surgery, to facilitate biopharmaceutical development, to study the structure–function relationships of genomes, to generate transgenic cell lines and animals, and to produce both food and biofuels (Saha *et al.*, 2018).

**Figure 3:**
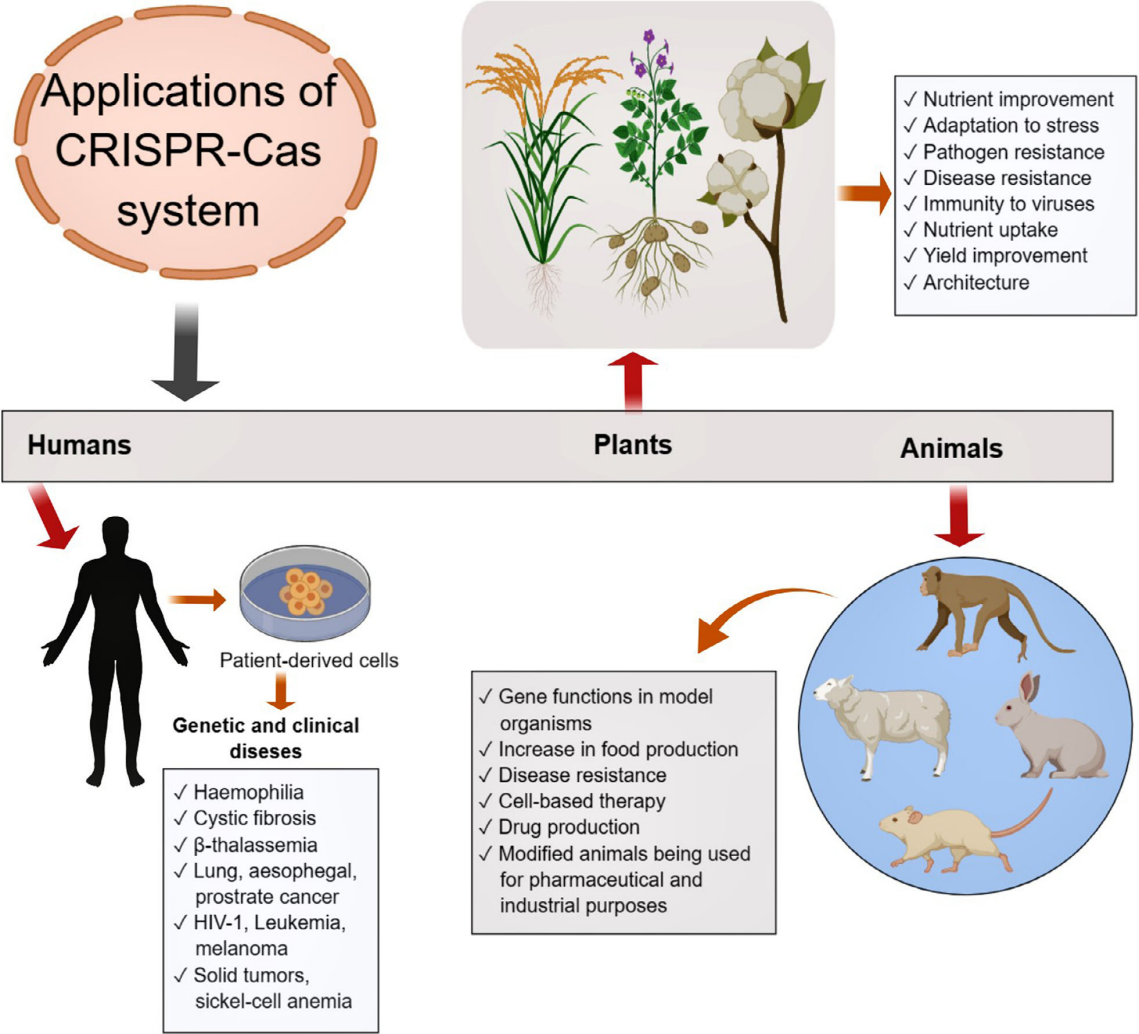
Applications of CRSPR‐Cas system in clinical and genetic diseases and trait improvements in plants and animals.

Because of its versatility, the CRISPR‐Cas system has also therapeutic potential (Li *et al.*, 2015). It can be employed to correct modifications in numerous genetic disorders (Figure 3), such as haemophilia, cystic fibrosis, β‐thalassemia, hereditary tyrosinaemia type I and Duchenne muscular dystrophy (Long *et al.*, 2014; Sürün *et al.*, 2018; Xie *et al.*, 2014; Yin *et al.*, 2014). CRISPR‐Cas9 has enabled scientists to repair mutations in the *cftr* locus that causes cystic fibrosis in cultured intestinal stem cells from cystic fibrosis patients (Hille and Charpentier, 2016; Schwank *et al.*, 2013). Moreover, CRISPR‐Cas9 offers considerable promise for curing a range of other human diseases (Figure 3). For example, several clinical trials around the world are utilizing CRISPR‐Cas9. These trials focus on multiple myeloma, lung, oesophageal, bladder and prostate cancer, HIV‐1, human papilloma virus, leukaemia, melanoma, solid tumours, gastrointestinal infection, sickle cell anaemia and other diseases (Brokowski and Adli, 2019; Cyranoski, 2016). Up till May of 2018, in China, the genes of at least 86 individuals were altered as part of clinical trials (The, 2018). In addition, CRISPR‐Cas9 repaired the genetic lesion that causes mice to suffer from hereditary tyrosinaemia (a genetic disease causing severe liver damage) (Yin *et al.*, 2014). This technology allows scientists to explicitly determine the biological functions of genes. For example, scientists are able to determine whether a gene contributes to a metabolic or pathogenic process by engineering a loss‐of‐function mutation in that gene and analyse the phenotype of the resulting mutant. Using this approach, genes that are involved in tumour growth (Chen *et al.*, 2015) or that convey susceptibility towards bacterial toxins (Hille and Charpentier, 2016; Koike‐Yusa *et al.*, 2014) are identified.

In addition to biomedical research, the agricultural and food sciences have considerable interest in using this technology to increase the supply and quality of food (Eş *et al.*, 2019). The first agricultural study of the CRISPR‐Cas9 system reported its higher potential to edit the rice genome (Feng *et al.*, 2013). This study revealed the usefulness of this technique in agriculture and thus encouraged the use of this technology to genetically modify other sources of food. In addition, the improved designs for sgRNAs increased the efficiency of CRISPR‐Cas genome editing in many high nutritional value crops and other plants, such as maize (Liang *et al.*, 2014), wheat (Wang *et al.*, 2014), rice (Li *et al.*, 2020; Miao *et al.*, 2013), soybean (Bai *et al.*, 2020; Chilcoat *et al.*, 2017), sweet orange (Jia and Wang, 2014), tomato (Brooks *et al.*, 2014), rapeseed (Zheng *et al.*, 2020), ryegrass (Zhang *et al.*, 2020) and *Physcomitrella patens* (Yi and Gosima, 2019). Based on these promising results, a broad range of CRISPR‐Cas9 applications seem possible in crops.

In crops, the CRISPR‐Cas system is effectively utilized to improve important genetic traits (Figure 3), for instance stress tolerance, disease resistance, nutrient uptake, yield and architecture. It has been effectively employed to enhance plant immunity to viruses. For example, CRISPR‐Cas system was used to engineer *Solanum lycopersicum* and *Nicotiana benthamiana* plants to develop resistance against tomato yellow leaf curl virus (TYLCV) (Tashkandi *et al.*, 2018). Recently, Aman *et al. *(2018) employed CRISPR‐Cas13a in *N. benthamiana* to cleave the single‐stranded RNA (ssRNA) of turnip mosaic virus (TuMV). Although it targeted different sites of the viral genome, the crRNAs exhibited the most effective interference and viral genome degradation activities when they targeted the sequences that encode HC‐Pro.

CRISPR‐Cas9 has shown promise as a tool for engineering the resistance to several diseases in plants. CRISPR‐Cas9 was used to produce bread wheat that is immune to powdery mildew by mutagenizing the *TaMLO‐A1* and *TaMLO‐B1* genes (Wang *et al.*, 2014). Another study produced allotetraploid cotton that is resistant to *Verticillium wilt* utilizing two sgRNAs—GhMYB25‐like‐sgRNA1 and sgRNA2 (Li *et al.*, 2017). Indeed, using the CRISPR‐Cas genome editing machinery for editing host genome to confer resistance seems to be the most effective approach. Consistent with this idea, in tomato and *A. thaliana*, CRISPR‐Cas improved the resistance against important pathogens, such as *Phytophthora capsici, Phytophthora syringae* and *Xanthomonas* spp. (de Toledo Thomazella *et al.*, 2016). In addition, a plasmid‐free CRISPR‐Cas9 system combined with ribonucleoproteins (RNPs) was used to fight the rice blast fungus *Magnaporthe oryzae,* which significantly damages rice production globally (Foster *et al.*, 2018). This system seems simple and rapid and thus can help to increase progress in developing plants that resist these plant pathogens.

In addition to increasing biotic stress tolerance, the CRISPR‐Cas9 toolbox can be used to improve the nutritional value of plants. The CRISPR‐Cas9 machinery has been used to increase the nutrient content of potato by knocking out the granule‐bound starch synthase (GBSS) gene (Andersson *et al.*, 2017). Similarly, CRISPR‐Cas9 mutagenized the *SBEI* and *SBEIIb* genes in rice. This modification increased the levels of amylose and resistant starch, yielding rice grain with improved nutritional value (Sun *et al.*, 2017). Recently, pYL CRISPR‐Cas9 multiplex vector system was developed in tomato to manipulate a particular metabolic pathway and yield larger quantities of γ‐aminobutyric acid (GABA) (Li *et al.*, 2018). Moreover, the other CRISPR‐Cas systems are increasingly being used in major crops as well, such as CRISPR‐Cpf1 has successfully been used in cotton (Li *et al.,* 2019a), maize (Lee *et al.*, 2019) and rice (Tang *et al.*, 2018; Xu *et al.*, 2018). Base editing systems using modified CRISPR‐Cas9 system have been developed in cotton to create single base modification. This system can be used to create precise changes in amino acids through changing a specific single base or develop a gene knockout via introducing a premature stop codon (Qin *et al.*, 2019). The base editing system has also been applied in different plants, such as wheat, maize, rice and tomato (Ren *et al.*, 2018; Shimatani *et al.*, 2017; Zong *et al.*, 2017).

The possible applications of modified CRISPR‐Cas systems for precise improvement of commercially important crops by transgenic and non‐transgenic processes are now increasing. Importantly, the combination of CRISPR‐Cas system and plant tissue culture has now enabled the direct introduction of desired genetic alterations into commercially significant genotypes. This approach can introduce genetic changes directly into the relevant lines with increased speed and specificity relative to traditional breeding methods (Adli, 2018).

## Ethical considerations for the CRISPR‐Cas system

Over last two decades, biotechnology has developed rapidly and has enabled us to develop scientific tools that years ago seemed like science fiction. Genome editing, particularly CRISPR‐Cas system, presents one of the foremost examples of this sort of advance to biotechnology because it allows us to structurally modify genetic background of living organisms including humankinds (de Lecuona *et al.*, 2017). Despite the CRISPR‐Cas system allows us to effectively manipulate genomic DNA (Lyon, 2017), there are a wide range of moral, ethical, political and scientific concerns that may lead to a moratorium that would paralyse this kind of research. Therefore, it is essential to identify the problems, elucidate concepts and promote discussion among all of the concerned stakeholders, such as universities, societies, and science and technology systems to articulate an optimal ethical and legal framework (de Lecuona *et al.*, 2017; Lyon, 2017).

In this regard, the legal, ethical and biomedical aspects of CRISPR‐Cas9 were discussed by a group of CRISPR‐Cas9 developers, scientists and ethicists during Napa Valley meeting in 2015 (Baltimore *et al.*, 2015). After this meeting, widespread discussions were also entreated. The United States (US) National Academies of Sciences, Engineering and Medicine (NASEM) invited the Chinese Academy of Sciences and the United Kingdom's (UK) Royal Society to participate in an International Summit on Human Gene Editing in order to discuss how, when and where to apply this technology in humans (Brokowski and Adli, 2019). Soon after this meeting, the Nuffield Council on Bioethics called for evidence on the ethical issues evolving from these biological techniques that allow us to precisely alter the genes of living cells and their applications in biomedicine, reproduction, plants and animals, industrial biotechnology and ecology (Mulvihill *et al.*, 2017; Nuffield Council on Bioethics, 2016).

## Basic pre‐clinical and clinical research

Currently, CRISPR‐Cas technique is a feasible option for genome modification in wide range of organisms including humans, where this technique has been used for treating or preventing serious genetic diseases (Hildebrandt and Marron, 2018). CRISPR‐Cas technique can be highly beneficial in basic and pre‐clinical settings to clearly understand and further improve the technology itself in order to be applicable in clinical research. But a major concern about the CRISPR‐Cas technique is its possible application in human embryo germ‐line editing because our understanding of human embryo status is insufficient and it is very difficult to declare whether and precisely when it achieves the ‘personhood’ (Miklavcic and Flaman, 2017). Thus, the moral status of embryo itself prevents any kind of modification in supernumerary embryo. However, the genomes of human embryos were first edited with CRISPR‐Cas9 by Chinese scientists in 2015. This study on non‐viable tripronuclear embryos revealed off‐target mutations and mosaicism (Liang *et al.*, 2015; Rossant, 2018), which heightened community awareness and sparked numerous responses from organizations and groups around the world to develop ethical polices to address germ‐line gene editing.

Various countries have developed regulatory laws or guidelines for human germ‐line gene editing for reproductive purposes. These ethical guidelines vary widely around the world such as ‘restrictive’ in the United States, ‘legal prohibition’ in the UK, ‘prohibition by guidelines’ in Japan, China, Ireland and India, and ‘ambiguous’ in Russia, Argentina, South Africa, Chile, Slovakia, Peru, Colombia, Iceland and Greece (Ishii, 2017, 2017). Additionally, the modification of human embryo beyond 14 days of its development is ethically prohibited in many countries (Hyun *et al.*, 2016; Nuffield Council on Bioethics, 2017). Taken together, the ethics of germ‐line genome editing lack societal consent globally ranging from ‘ban’ in some countries to ‘outlawing in any circumstances’ in others (Coller, 2019). In International law however, germ‐line genome editing is prohibited and highly discourages by the UNESCO Declaration on the Human Genome and Human Rights as well as the Council of Europe Convention on Human Rights and Biomedicine (Dickenson and Darnovsky, 2019). However, human genome modification proceeded anywhere in the world should follow international agreements and norms in order to protect human values that is of utmost importance in human genome experimentation, as divergence in cultural and religious standards will possibly prevent to attain a global consent on uniform rules and regulations. Addressing these concerns, NASEM report has recommended certain principles: promoting the well‐being, fairness, respect for persons, responsible science, due care, transparency and transnational cooperation (Coller, 2019).

The NASEM report provides an extensively analysed set of guidelines considering wide‐ranging issues of human genome editing. The committee has advocated the somatic genome editing but has not allowed genome editing applications for any kind of enhancement (NASEM, 2017). Although not allowed currently, the committee has concluded that the modification of the germ‐line in order to create a new person who might possibly transfer edited genome to future generations could be allowed under specific conditions: (i) germ‐line editing can be applied to treat serious genetic disorders, if no alternative exists and should proceed with caution under strict oversight and public input, (ii) the genome editing of somatic and germ cells for enhancement purposes cannot be performed at this time, (iii) genome editing can be performed for the purpose of basic research and (iv) somatic genome editing can be performed to treat serious diseases. Both (iii) and (iv) can be carried out under existing regulatory processes. The NASEM report implies that if the technical and safety concerns are well understood, a clinical trial on germ‐line editing may be started (Rossant, 2018).

In addition, Nuffield Council on Bioethics report issued in 2018 supported germ‐line genome editing that could be permitted under certain conditions, such as ‘there are moral reasons to continue with the present lines of research and to secure the conditions under which heritable genome editing would be permissible’ (Dickenson and Darnovsky, 2019; Nuffield Council on Bioethics, 2018). However, the principles of Nuffield Council on Bioethics as well as NASEM downplay many technical, moral and societal concerns about reproductive genome editing to be accepted (Dickenson and Darnovsky, 2019; Peters, 2019). Though it has been understood that clinical applications of germ‐line gene editing at this time cannot be performed anywhere in the world, the report on gene‐edited babies outreaching ethical borders to threatening the future of human species evidences that these principles are not yet adequate enough to proceed germ‐line gene editing (Lander *et al.*, 2019).

After the report of gene‐edited babies, a moratorium was called to establish an international framework (Lander *et al.*, 2019). Considering wide‐ranging issues of the technology, the moratorium presented some worldwide recommendations for germ‐line gene editing: (I) retaining their own rights, the nations should voluntarily pledge to not allow the application of clinical germ‐line editing until it meets certain conditions, (II) a specific timeframe should be fixed during which no any clinical application of germ‐line gene editing should be permitted, (III) before allowing specific germ‐line gene editing, the countries should provide public notice of performing application along with consulting the purpose of the application in international discussion, (IV) the application should be justified by transparent evaluation, (V) extensive societal consensus should be established in the nation to define the appropriateness of the germ‐line genome editing and (VI) the countries may not choose the same path, at least they should commit to proceed openly with due respect to the human issues that would ultimately affect the entire species.

In addition to this, a satisfactory risk–benefit analysis is insufficient for an ethical trial. A strategy to monitor intergenerational and other unique ethical issues is necessary for the safety of future clinical use of germ‐line genome editing. Furthermore, safety of dignity, privacy and welfare of the participants under study are of the utmost importance. No extent of therapeutic potential can justify proceeding with human experimentation until these protections are secured (Cwik, 2017). Thus, a borderline should be drawn for every kind of genetic enhancement; otherwise, nations might someday proceed limited or extensive application of enhancement.

## Agriculture

CRISPR‐Cas system has been the preferred method for crop improvement because it is a robust, quick and accurate method for genome manipulation. The commercial use of new breeds of genome‐edited crops has raised moral, regulatory and biosafety concerns because they affect the living condition of present and future moral entities, including the non‐human environment (Globus and Qimron, 2018). Although CRISPR‐Cas can be used to produce both the cisgenesis and transgenesis, the present debate on acceptance of this system in agriculture has raised attention on cisgenic products (Palmgren *et al.*, 2015). In this scientific context, the technique per se is morally neutral (Weigel, 2017). The menace caused by novel animals or plants for nature‐identical genetically modified organisms (nGMOs) is likely same that of conventionally bred counterparts because they could be the outcome of natural evolution (Zilberman *et al.*, 2018). Though the commentaries on the new technology have highlighted unexpected risks of off‐target editing and possible misuse of the technology, those discussed above (Li *et al.,* ,2019b; Steinbrecher, 2015; Wang *et al.*, 2019a), the main reason to turndown genetic engineering is the risk to human health and environment due to crossing reproductive limits seems now obsolete. Based on a utilitarian viewpoint, the manipulation of CRISPR‐Cas without the insertion of a transgene is comparatively less risky compared to previous GM techniques; thus, it is morally more acceptable (Bartkowski *et al.*, 2018). Because GMOs have been generated through transgenic approach by the insertion of an exogenous gene into the host genome (Li *et al.,* 2019b), and in the case of genome editing, such as CRISPR‐Cas systems (using NHEJ repair mechanism), genetic alteration is mainly based on small indels of nucleotides within the endogenous target gene. Such modifications are like the natural variations as well as those generated by chemical and physical mutagenesis but are generated with high specificity and efficacy through genome editing process (Globus and Qimron, 2018; Huang *et al.*, 2016; Li *et al.,* 2019b). Additionally, the expression cassettes introduced to enable plant genome editing can be removed from the progeny through genetic segregation, or these are never delivered in the genome at all when transient expression approach or ribonucleoprotein complex is used. Therefore, genetic properties of genome‐edited crops are different from traditional GMOs but are considerably similar to the crops produced by natural or mutagenesis approach. Thus, genome‐edited crops could be considered to have low risk to human safety (Li *et al.,* 2019b; Schulman *et al.*, 2019).

Besides the debate of principle‐based acceptance of biotechnology, the ethical discussion about CRISPR‐Cas in plant breeding has been focused on common ethical critique of modern agriculture along with its ecological and societal acceptance. The ethical issues raised by CRISPR‐Cas‐based genome editing crossed the boundaries of satisfactory outcomes and side effects. Applications of CRISPR‐Cas9 raise fundamental societal interrogations about justice as well as intra‐ and intergenerational fairness. Nonetheless, in pluralist societies there should be a societal debate on guiding standards about the development and the use of any novel technology (Bartkowski *et al.*, 2018).

## CRISPR‐Cas9 patent landscape

Compared to other genome editing techniques like ZFNs and TALENs (Klug, 2010; Li *et al.*, 2010; Perez Rojo *et al.*, 2018), CRISPR was initially developed in academic research institutions (Brinegar *et al.*, 2017). Doudna and Charpentier in 2012 published their first paper on CRISPR‐Cas9 and initiated their patent application on DNA editing using CRISPR‐Cas9 (Jinek *et al.*, 2012). By end of the same year, another research group led by Feng Zhang introduced another patent application demonstrating the use of CRISPR‐Cas9 in mammalian cells (Cong *et al.*, 2013; Ledford, 2016). Their article published in 2013, which started controversy between two groups about the intellectual property associated with CRISPR‐Cas9 (Perez Rojo *et al.*, 2018).

After several years of controversy, the US Supreme Court in 2013 set a rule that human genes cannot be patented because the DNA is a ‘product of nature’ (Joly and Tonin, 2014; Sherkow and Greely, 2015). The decision further indicated that there would be jurisdictional differences and questions associated with the novelty of the CRISPR system. For example, ‘Is it novel? An invention or merely nature discovered?’ However, the CRISPR may not be regarded natural product because it can be altered to function in animal and human cells where it does not function naturally (Beale, 2016). To some, it may not be clear that CRISPR was invented because it was discovered in a bacterial system. But its discovery and the subsequent steps required to develop a well‐functioning genome editing tool remained the subjects of patent claims (Mulvihill *et al.*, 2017).

Despite this conflict, the US Patent and Trademark Office awarded the first CRISPR‐Cas9 patent rights to Zhang based on the ability of his system to edit DNA and the capacity to control and modify CRISPR for the manipulation of animal and human cells—a cellular system where CRISPR is not existing naturally. However, Doudna then filed an interference claim against Zhang to determine that who was the first to discover CRISPR‐Cas9 and modify it to function in a nonbacterial cell (Mulvihill *et al.*, 2017). In short, these patent clashes have developed a complex and competitive environment and that will probably divert attention from the public good (Ledford, 2016). Based on previous experience with patent issues (e.g. the patents on stem cells, DNA sequences and genetic variants manipulated to serve as diagnostic tools), the current situation may negatively affect the development, research and access to CRISPR techniques.

## Global regulatory policies for genome editing with the CRISPR‐Cas system

The early genome editing studies on human embryos prompted deliberations on ethics, morality and safety, insisting researchers to stop performing genome editing experiments on human germ cells using CRISPR‐Cas9 for clinical purposes until safety concerns are resolved and the ethical and societal implications of specific germ‐line alteration could be extensively discussed publically among wide‐ranging societal groups (Baltimore *et al.*, 2015; Olson *et al.*, 2016). The NASEM report has broadly discussed the principles of clinical applications along with regulatory framework for genome editing (NASEM, 2017). Nonetheless, the regulatory criteria for these applications are still unclear in some respects. In addition, the regulatory criteria for genome‐edited plants by CRISPR‐Cas are poorly discussed internationally that how edited plants should be governed? Whether edited plants/crops should be regulated as GMOs (genetically modified organisms). Thus, deliberative discussions continue worldwide on how genome editing might be different from other biotechnologies in principle and in practice (Fears and Ter Meulen, 2017).

**Figure 4:**
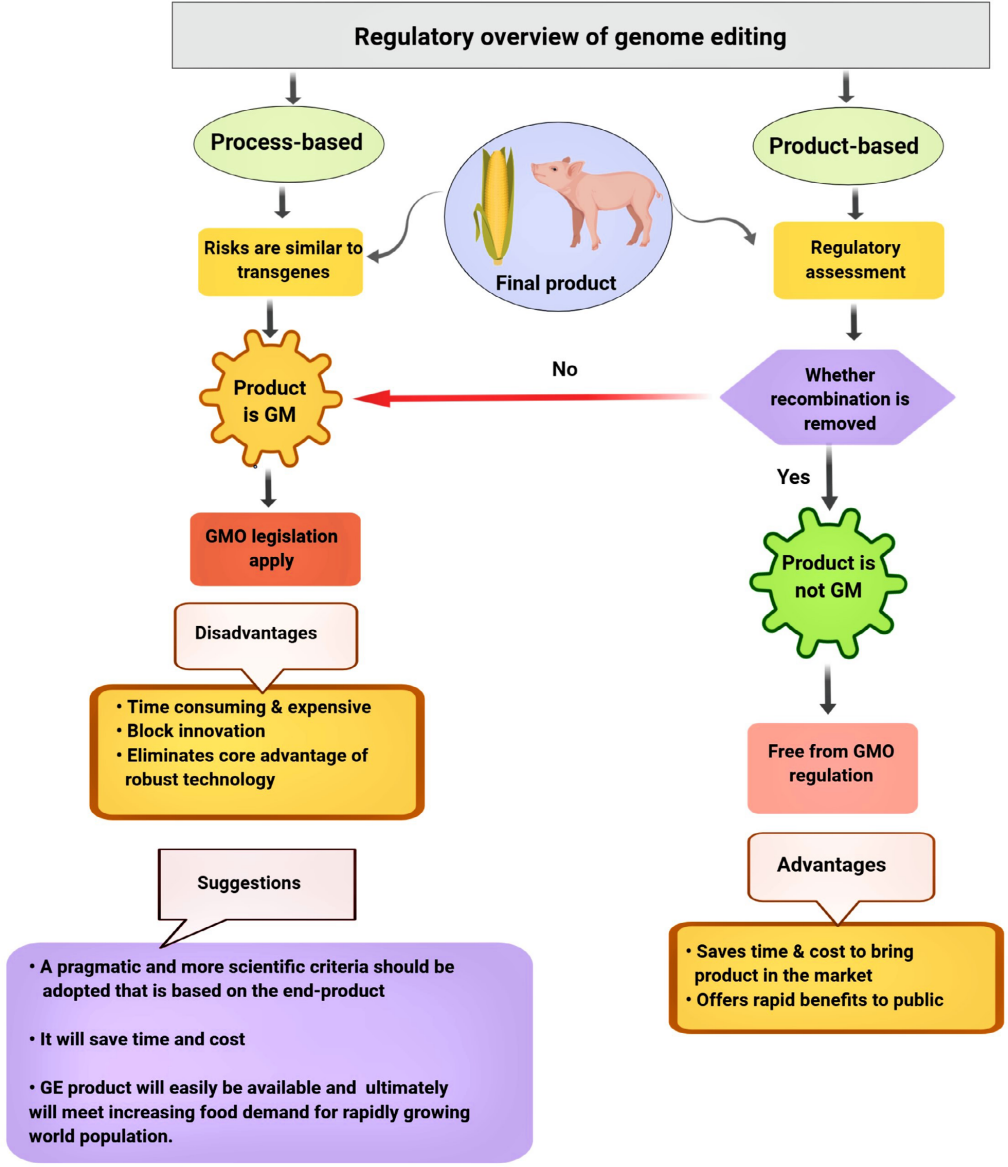
Global regulatory overview of genome editing. Currently, one of two regulatory systems has been used in different nations. Process‐based: Some countries (European Union and New Zealand) regulate genome‐edited end products based on the process used, which leads edited products to GMO regulation. Product‐based: United States and many other countries evaluate genome‐modified end products based on the characteristics of end product. If, the product is transgene‐free, it does not fall under GMO scope, but, if the product contains any transgene, it is regulated under GMO regulation. GM: genetically modified. GMO: genetically modified organism. GE: genome‐edited.

Since research developments are being translated into novel crops, these scientific developments create new challenges for global regulatory authorities. For example, to what extent should previous legislation governing GMOs apply to plant or food products developed using genome editing (Araki and Ishii, 2015)? There are two important issues for countries as they decide how to regulate genome‐edited plants. First, can there be exclusions from regulation for certain categories of genome‐edited plants based on process (techniques used to produce the plants) or properties (whether it is a low‐risk product that can be excluded from regulatory oversight)? (Figure 4). Second, for genome‐edited plants subjected to regulation in a specific country, what is the necessary safety data that must be provided to a regulatory authority? The latter question is particularly important for genome modifications that fall into either the SDN‐1 (site‐directed nuclease 1) or SDN‐2 categories (Zannoni, 2019). The worldwide social and regulatory system on GM crops (Table 1) is complex with distinct regulatory frameworks in place (Davison and Ammann, 2017). The two main types of frameworks, either process or product‐oriented framework (Figure 4), have been used (Ishii and Araki, 2017).

**Table 1:** Regulatory policies of different nations for plant genome editing including CRISPR‐Cas9

Country	Agency	Policy	Criteria	References
European Union	EFSA	GMO	Process‐based	Jouanin *et al. *(2018)
United States	USDA‐APHIS	Non‐GMO	Product‐based	Kleter *et al. *(2019)
Canada	CFIA	–	Product and novel trait	Schuttelaar (2015)
Argentina	CONABIA	Non‐GMO: if final product proved to be non‐transgene. GMO: if final product is transgene	Product‐based	Gao *et al. *(2018)
Australia	OGTR	Non‐GMO: if final product proved to be non‐transgene.	–	Mallapaty (2019); Tchetvertakov (2019)
China	–	GMO: if final product is transgene	Case‐by‐case	Gao *et al. *(2018)
New Zealand	EPA	GMO	Process‐based	Fritsche *et al. *(2018)
Japan	Ministry of Environment	Non‐GMO: if final product proved to be non‐transgene. GMO: if final product is transgene	–	Zannoni (2019)
Brazil	CTNBio	Non‐GMO: if final product proved to be non‐transgene.	Case‐by‐case	Eriksson *et al. *(2019)
Sweden	SBA	Exempted from GMO: if foreign DNA is not integrated	Case‐by‐case	Pfeiffer *et al. *(2018) and Eriksson (2018)
France	HCB	Exempted from GMO: if exogenous gene is removed GMO: if exogenous gene is not removed	Product‐based	Pfeiffer *et al. *(2018)
Chile	Forestry and Agricultural Protection Division of SAG	Exempted from GMO: if lacking new combination of genetic material GMO: if new combination of genetic material has occurred	Case‐by‐case	Eriksson *et al. *(2019)

In different nations, varying polices and criteria have been used to regulate genome‐edited plants. Most of the nations have exempted edited plants with non‐transgenes from GMO legislation, and few nations still consider edited plants as GMO. Similarly, edited plants have been assessed based on the end product or the process used. GMO, genetically modified organism; EFSA, European Food Safety Authority; USDA‐APHIS, US Department of Agriculture‐Animal and Plant Health Inspection Service; CFIA, Canadian Food Inspection Agency; CONABIA, National Commission Advisor in Agricultural Biotechnology; OGTR, Office of the Gene Technology Regulator; EPA, Environmental Protection Authority; CTNBio, Brazilian National Biosafety Technical Commission; SBA, Swedish Board of Agriculture; HCB, High Council for Biotechnology and SAG, Agricultural and Livestock Service.

In plant genome editing, site‐directed mutagenesis has been frequently performed without introducing transgenes (Araki and Ishii, 2015). At present, to avoid undesirable DNA integration and bypass current GMO regulations, newer genome editing techniques (such as CRISPR‐Cas system) that do not introduce DNA into cells are now being developed and optimized for use in crops, such as wheat and rice (Li *et al.*, 2020; Liang *et al.*, 2017; Malnoy *et al.*, 2016). Several countries, for example Canada, the United States and Argentina, have decided that legislation that regulates GMOs will not regulate plants produced by using genome editing techniques if they do not contain foreign DNA (Waltz, 2018). On the contrary, the European Court of Justice (ECJ) in case C‐528/16 has declared that the organisms produced through mutagenesis techniques are regulated as GMOs and are subjected to the GMO directive, because in the process of mutagenesis, the genetic material of the organism is altered in a way that does not occur naturally (ECJ, 2018a).

If the CRISPR‐Cas technology is regarded as a variant of traditional genetic engineering with GMO production as the final result, it seems logical to apply the current process‐oriented EU legislation to the technology. This poses a disadvantage to biotech companies that use the technology, because of the additional bureaucratic burden and financial costs entailed by such legislative recognition, such as the need to get marketing permission and labelling of the products (Medvedieva and Blume, 2018). Enabling a broad progress and use of crops developed by genome editing will need additional clarification of regulatory principles for genetically modified (GM) crops. Therefore, a resolution should be made for present bipolar regulatory criteria (it encompasses both the product and the process of bioengineering under regulatory decisions) to overcome misunderstanding among regulators, civil society and product developers regarding the extent and nature of concerns related to genome editing crops (Wolt, 2017).

## Regulatory policies for genome editing in the United States

In the United States (US), the regulatory situation is considerably different, where the regulatory policies of biotechnology products are governed through a Coordinated Framework. The main agencies functioning within the Coordinated Framework for the evaluation of bioengineered crops involve the Environmental Protection Authority (EPA), the US Department of Agriculture (USDA) and the Food and Drug Administration (FDA). These agencies address the effects of bioengineered crops on the environment, agriculture and human health, respectively (Wolt, 2017). In addition, the USDA Animal and Plant Health Inspection Service (APHIS) is especially charged with regulating the genome‐edited crops. APHIS applies the Regulated Articles Letters of Inquiry to determine the regulatory standard of proposed products via the ‘Am I Regulated?’ portal (https://www.aphis.usda.gov/aphis/ourfocus/biotechnology/ami‐regulated; Wolt, 2017). Companies are required to access this portal and to submit their enquiries in order to know whether their engineered organisms/products fall outside of the USDA’s scope (Waltz, 2018).

Responses to these inquiries indicate that genome editing crops that contain simple indels of a limited number of bases and that lack transgenic elements in the end product are not regulated by the USDA (Wolt *et al.*, 2016). However, the USDA appears to remain interested in regulating genome editing crops containing small templates or directed transgene insertions (Camacho *et al.*, 2014). Thus, a one‐size‐fits‐all criteria cannot be applicable for regulation of genome editing crops by the USDA. Therefore, the EPA, FDA and USDA were directed to consider an update to the Coordinated Framework. Additionally, the agencies were directed to work along an interagency Biotechnology Working Group to establish a long‐term framework to regulate future products of biotechnology (Executive Office of the President, 2015; Wolt, 2017).

Moreover, the APHIS proposed extensive revisions (82 FR 7008, 1/19/2017) to its policy of not regulating crops with simple genetic changes that could be obtained using either CRISPR‐Cas9 or using both radiation‐ and chemical‐based mutagenesis, such as deletions—irrespective of size—and single base pair substitutions. According to a decision announced by the USDA in 2016, the use of CRISPR‐Cas technology does not need any additional regulation because the final product does not contain alien DNA from plant pests, such as viruses and bacteria (Smyth, 2017; Waltz, 2016). Therefore, this decision implies that plant cultures produced using the CRISPR‐Cas technology are beyond the scope of its regulations developed for GMO products because they do not contain foreign DNA and cannot be distinguished from cultures produced by traditional selection methods (Medvedieva and Blume, 2018). The APHIS has made several announcements indicating that it does not require risk assessments for transgene‐free genome editing plants (Ishii and Araki, 2017). In January of 2017, APHIS proposed to change the regulatory mechanisms for GM plants to abolish the application of legislation concerning plant pests to ‘gene‐edited’ plants. Thus, the main regulatory bodies of the United States have not reached a consensus on whether the laws concerning human health and environmental risks should be applied to gene‐edited organisms (Medvedieva and Blume, 2018).

Recently, the USDA declared that they will not regulate plants edited using CRISPR‐Cas system and that these plants can be cultivated and sold—as it gave a free pass to *Camelina sativa* (false flax). Moreover, in October 2017, the agency stated that it will not regulate a CRISPR‐Cas9‐edited soybean variety with enhanced drought‐tolerance. This laissez faire approach will allow biotechnology companies to bring new varieties of plants into the market years earlier and for millions of dollars less than plants that the USDA classifies as GMOs. Edited drought‐tolerant soybean and false flax produced by scientists by the USDA’s research arm are two of at least five CRISPR‐Cas9 modified organisms that sidestepped the USDA’s regulatory purview in the last three years (Waltz, 2016).

The US regulations and policies are mainly technical and are based on both the engineered traits (Table 1) and the intended use of the products (NASEM, 2016). Basically, they consider the risks posed by the traits of new products under its coordinated regulatory framework. Other implications, such as socioeconomic, moral and cultural issues, have little to no impact on these regulations and policies (Globus and Qimron, 2018; Kleter *et al.*, 2019). The APHIS assesses whether new genetically edited plants can be characterized as either plant pests or noxious weeds. If new varieties of crop plants produced with CRISPR‐Cas9 are without these characteristics, they are not regulated by the USDA (https://www.aphis.usda.gov/aphis/ourfocus/biotechnology/amiregulated/Regulated_Article_Letters_of_Inquiry; Kleter *et al.*, 2019).

The classification of plants produced using these techniques as GMOs may depend on whether a process or end product is examined. Many scientists and potential authorities worldwide have suggested that the regulation should proceed from a process‐based to a product‐based approach (Globus and Qimron, 2018). Meaning, rather than evaluating the process—that is long and expensive—risks should be evaluated by considering the end products, after surveying off‐target mutations. Furthermore, the USDA opinions also indicate that they do not regulate particular mutations caused by TALENs, ZFNs, meganucleases and CRISPR that induce the NHEJ pathway (Wolt *et al.*, 2016). Taken together, these trends provide evidence that the CRISPR‐Cas technique could be employed to develop plants that are not considered GMOs in the United States.

## Canadian regulatory perspective

Canada has developed a regulatory framework for biotechnology that is based on ‘the product and its novel trait’ and not by the procedure used to produce the plant (Gao *et al.*, 2018; Schuttelaar, 2015). The Canadian Food Inspection Agency (CFIA) defines new plants based on their novel traits and not the process used to produce these plants (Schuttelaar, 2015). Thus, plants with novel traits obtained from a process that includes gene editing, transgenesis, mutagenesis or conventional breeding are subjected to the same regulatory approval process, which is controlled by CFIA in cooperation with Health Canada (Gao *et al.*, 2018). The sale of foods developed from plant new traits (PNTs) is regulated by Health Canada through a mandatory requirement of pre‐market notification (Wolt *et al.*, 2016). Thus, the herbicide‐tolerant variety of canola was approved by CFIA and Health Canada in 2014 as the first commercial crop produced by genome editing (Jones, 2015).

## Regulations for GE in Europe

The potential application of genome editing as a crop improvement tool remained the subject of long‐term debate within the EU research and regulatory community (Pauwels *et al.*, 2014). Initially, European Commission argued that the herbicide‐resistant canola produced using genome editing without providing any legal guidance. The long timeline of the regulatory and legal deliberation for this product was influenced principally by the subtleties of the EU GMO definition, which is subject to interpretation. One interpretation is that these products should be regulated based on the process used to generate the product. Another interpretation is that these products should be regulated based on the nature of the product that was developed (Sprink *et al.*, 2016). Thus, it is the need to adopt regulatory policies that are more practical and scientifically defendable because they are based on the nature of the final product (Wolt *et al.*, 2016). In Europe, regulators have mainly remained mute on the ultimate regulatory fate of gene‐edited crops (Wolt, 2017).

In the European Union and the UK, current policies and GMO guidelines continue to include animals, plants, fungi and microorganisms modified using genome editing under the GMO umbrella (Table 1; Spicer and Molnar, 2018). Thus, the foods produced by GM must be properly labelled by law (Globus and Qimron, 2018). In the EU, European Food Safety Authority (EFSA) is accredited to scientifically review the safety and environmental impact of GMOs. The scientific agencies of member states mainly coordinate with EFSA to develop scientific viewpoints. But the final decision on whether to regulate genome editing crops is made by the European Commission (EC). Although a crop approved by the EC will probably not be cultivated in all of the member states, the intense public controversy over genome editing crops in particular member states prevents the EU from using GM crops (Globus and Qimron, 2018).

Many states within the EU have developed their own policies favouring a non‐GMO label for genome editing crops (Wolt, 2017) and are thus not waiting for an official decision from the EU or the European Court of Human Justice (ECHJ). These states include Finland and Sweden. Moreover, Germany and the Netherlands are also approaching a ruling in favour of a non‐GMO label for crops produced using genome editing (Spicer and Molnar, 2018). All of these countries that are moving ahead by making their national recommendations have demonstrated that they will defer to EU rulings if and when they are made. Thus, it is the need to completely redefine the concept of GMOs and their associated risks and regulations. Such as, a future definition and risk assessment process may work together on a particular type of product or on a case‐by‐case basis, taking the pros, cons and risks into account (Spicer and Molnar, 2018; Stilgoe *et al.*, 2013). For example, gene‐edited plants which are similar to conventional varieties, anticipating their possible consequences for food security, environmental safety and economic issues, the ‘innovation principle’ can be followed. Where, relevant risks will be assessed based on a case‐by‐case basis, which will enable to get advantage from a gene‐edited product while complying with related risk management. Thus, it will develop a suitable approach to regulate food security, environment protection and economy (Jouanin *et al.*, 2018).

The complexities of regulatory policies develop hurdles for genome editing techniques, especially those using gene knockout or nucleotide variants. These techniques are by nature same as the use of induced mutations or spontaneous variants in conventional breeding, offering the advantage that only desired change is introduced. Thus, the crops edited by these techniques involve variations that exist within crop species or closely related wild species or that can be expected to produce with spontaneous mutation. Therefore, such crops should be considered similar to those generated through conventional breeding and should not be considered GMOs as foreign DNA is not stably introduced into the genome (Globus and Qimron, 2018; Huang *et al.*, 2016).

Many competent authorities EU, including EFSA, European Academies Science Advisory Council (EASAC) and the former Chief Scientific Advisor to the President of the EC, have supported that the products of new breeding techniques including genome editing, which do not introduce foreign DNA into the genome of a species, should not be regulated as GM (EFSA, 2015; Sprink *et al.*, 2016; EASAC, 2018) and should be considered with conventional breeding regulations or adopted regulations for genome editing products (Jouanin *et al.*, 2018; Whelan and Lema, 2015). In addition, Swedish Board of Agriculture (SBA) made interpretation for CRISPR‐Cas9‐edited plants which lack exogenous gene fall out of the GM legislation (SBA, 2015).

The European Plant Science Organization (EPSO) suggested that GMOs should not be defined only by the application of a particular technology. The EPSO suggests that a GMO should be defined as an organism that contains a new combination of genetic material that is not possible to obtain by mating and that results in unique recombination potential (EPSO, 2017). The EPSO also advocates for the position of the New Techniques Working Group, which concluded ‘most of the new plant breeding techniques such as genome editing do not fall under legal GMO definition as these techniques either fall out of the scope already established legislation or should be exempted because their products are not different from those obtained by traditional breeding’ (Duensing *et al.*, 2018).

Moreover, EASAC stated that policy concerns should be focused on the applications but not on the gene editing process itself as an emerging technique (EASAC, 2017). The regulation of applications should be evidence‐based, considering likely risks and benefits that is proportionate and considerably flexible to overcome future advances in the science. Further, the EASAC directed EU regulators to validate that the genome editing products, which are without a DNA from unrelated organism, fall out of the GMO legislation. In addition, the EASAC argues for complete transparency that reveals the process that was employed and the objective in the EU should be to regulate a particular trait or product instead of the technique used to produce the trait or product. These statements are consistent with the exemption of new genome editing technologies from regulations if genetic variations induced by genome editing techniques are indistinguishable or similar to the conventional breeding products, and if no novel product‐based risk is identified (Duensing *et al.*, 2018; Schulman *et al.*, 2019). In a product‐oriented regulatory approach, in fact, the regulations on plants produced by genome editing would be similar to the regulations on plants produced by mutation breeding. On the other hand, in a process‐oriented approach, such as the process employed by the EU, the genome editing plant is subjected to the process of GM risk assessment (Jouanin *et al.*, 2018).

However, regarding the organisms/plants produced using new techniques of mutagenesis, recently, the ECJ decided that according to the text of the Directive 2001/18/EC, 2001, such products should be regulated as GM (ECJ, 2018b; ECJ, 2018c). In fact, endorsing the use of GM regulations for the regulation of genome editing plants in Europe while simultaneously exempting plants obtained through mutation breeding is inconsistent. This is another example of an EU regulation based on process used, not on the product developed. This decision is based on the following precautionary principle: ‘A risk management approach, where if there is possibility that a given action or policy might cause harm to the environment or the public, and if there is no scientific consensus on the issue, the action or policy under question cannot be pursued. Furthermore, the scientific information becomes available and the situation should be reviewed’ (Jouanin *et al.*, 2018).

The ECJ classifies organisms generated by mutagenesis as GM, and therefore, these organisms must satisfy the obligations established by the GM Directive. ECJ takes the view that the risks caused by new mutagenesis techniques could be similar to the risks caused by transgenesis in GM plants. Therefore, the exemption of new mutagenesis techniques from the regulations required by GM legislation will violate the objective of the GM Directive and the precautionary principle described above. The completely process‐based approach of the ECJ considers the techniques employed and the end products obtained (Zannoni, 2019).

European GM regulations for genome editing plants are consistent with expensive ($35M) and time‐consuming (6 years) GM safety tests and administrative processes (Jouanin *et al.*, 2018; McDougall, 2011) with uncertain consequences because the final approval still remains a political decision. Moreover, GM regulation eliminates fundamental benefits of genome editing—a cheap, rapid and precise tool for generating high value‐added plants that can fulfil the requirements of consumers and society. Consequently, European researchers and research companies are moving to the United States to perform genome editing experiments (Burger and Evans, 2018). Thus, the regulation of genome editing plants as GM plants will block competitiveness, innovation and access to healthier food in Europe (Jouanin *et al.*, 2018).

In the UK, in February of 2015, the House of Commons presented advice on the faults of GMO legislation of the EU, consequences for future plant breeding techniques, products obtained from these techniques and trade issues. The House of Commons suggested that regulations based on process have failed to keep pace with technology and are potentially impeding innovation. The House of Commons further declared that the process‐based approach was too binding and therefore restricts progress on plant biotechnology in the UK. These remarks by the House of Commons initiated meaningful deliberations throughout Europe. Finally, the House of Commons advocated for a product‐based and not a process‐based approach for regulating crops produced by genome editing (House of Commons, 2015). In February of 2017, this advice was confirmed by the Parliamentary Office of Science and Technology (http://researchbriefings.parliament.uk/ResearchBriefin/Summary/POST‐PN‐0548; Pfeiffer *et al.*, 2018).

In February of 2016, the French ministries of Agriculture and of Ecology and the High Council for Biotechnology/Haut Conseil des Biotechnologies (HCB) published a report on its first step of reflections about the status of edited plants. The first proposal from the HCB argued that regulations for gene‐edited plants should be on the bases of characteristics of product instead of the techniques used to produce the edited plants. Further, the HCB stated that the new plant varieties developed by a process (SDN‐1) that does not require an assessment in line with Annex 1B of Directive 2001/18/EC (i.e. is not a GMO) must be subjected to only the conventional intellectual property (IP) and marketing authorization regulations. The criteria of SDN‐2 are similar to the criteria of SND1, except that the criteria of SDN‐2 allow breeders to introduce molecular signatures for traceability to support ownership claims (Pfeiffer *et al.*, 2018). However, according to SDN‐2, after molecular evaluation, if the modification is removed, the plant is exempted from risk assessment and is not treated differently than a plant produced through conventional breeding. In contrast, if a foreign gene remains, it will be subject to the GMO assessment regulations (HCB, 2016).

Trials for CRISPR‐Cas9‐edited Arabidopsis lines were carried by Swedish University of Agricultural Sciences and Umeå University independently, and asked Swedish Board of Agriculture (SBA) whether these plants are considered under GMO directive. The SBA declared that CRISPR‐Cas9‐edited plants in which exogenous DNA is not involved are exempted from GM legislation. Further, it was explained that if the foreign DNA exists within the edited plants, are regulated under GMO regulation (Eriksson, 2018) and these plants must be approved by the SBA because they are remitted to the European Directive for regulation (Pfeiffer *et al.*, 2018). In addition, the Swedish Government presented two alternative interpretations: firstly, the products developed with directed mutagenesis should not be directed under GMO regulation; and secondly, the same products might be considered GMOs but should be exempted from regulation (Eriksson, 2018). The SBA considers Category 2‐SDN‐1 as not under Directive 2001/18/EC and Category 3‐SDN‐1 as under Directive 2001/18/EC (Pfeiffer *et al.*, 2018). Thus, Sweden decided that the organisms obtained from mutagenesis that no longer carry recombinant nucleic acids or the organisms that are molecularly indistinguishable from naturally occurring mutations should—on a case‐by‐case basis—be exempted from GMO scope (Nature Eriksson, 2018; Editorial, 2017).

## Regulatory perspective in Australia

The application of genome editing techniques, including CRISPR‐Cas system, was restricted in Australia, and the use of this technology for research was governed by the same regulations those for conventional genetic modifications, which required permission from a biosafety committee endorsed by the Office of the Gene Technology Regulator (OGTR). Recently, Australia has reviewed its gene technology regulation in April 2019 by adopting the use of genome editing technology in plants and animals that does not introduce novel genetic material into the genome. The new ruling is approved for the genome editing techniques, which do not use templates and changes made are identical to the changes occurring naturally, thus do not cause additional risk to human health and the environment. However, the techniques that use template or introduce novel genetic material into the genome would inevitably be regulated by OGTR (Mallapaty, 2019; Tchetvertakov, 2019).

## Regulatory perspective in China

Recently, China has achieved a significant progress in genome editing technology that led to improvements in important crops, such as rice and wheat. The novel foods produced via genome editing need an updated risk analysis in China. To ensure food safety and to protect the agricultural‐products trade, China has established a regulatory framework for management and risk assessment of GM products that is regulated based on case‐by‐case analysis. In 2001, the State Council of China issued the ‘Regulation on Administration of Agricultural Genetically Modified Organisms Safety’, which defines GMO as: the plants, animals, microorganisms and their products with genetic structures that were altered through genome editing technology to be used in agricultural production or process. Thus, products derived via genome editing will be regulated under GMO regulatory system (Gao *et al.*, 2018) as interim policy. However, a working group was established within the National Biosafety Committee (NBC) in 2016 to deliver technical assistance on risk assessments for new technologies including genome editing but no formal regulations have been issued yet (Gao *et al.*, 2018). Present regulatory framework for gene‐edited crops in China subjects to a high level of scrutiny. Regulatory shift for gene‐edited plants those similar to natural or induced mutation has been urged by agricultural scientists in China to consider them as traditionally bred plants (Gao *et al.*, 2018; Xiaoyu, 2019). It will streamline heavy evaluation process and stimulate scientific research (Xiaoyu, 2019).

## Regulatory perspective in Brazil

The Brazilian National Biosafety Technical Commission (CTNBio) established a working group of experts in 2014, to establish a more updated normative for new breeding techniques (NBTs). The CTNBio’s Normative Resolution No. 16 (RN16) was approved in January 2018 (CTNBio, 2018). The normative assesses consultation based on a case‐by‐case system specifying that either a product crated by NBTs including CRISPR‐Cas9 will be recognized as a conventional or transgenic organism. According to normative, the progeny without the existence of recombinant DNA and/or RNA, the existence of genetic components that can be developed via conventional breeding, the existence of induced mutations that can also be created through older techniques and the existence of induced mutations that take place naturally are evaluated based on case‐by‐case analysis and are regarded conventional products/organisms (Eriksson *et al.*, 2019).

## Regulatory perspective in Argentina

Argentina has issued specific regulations regarding the products obtained through genome editing (Whelan and Lema, 2015). Resolution no. 173/15 of the Secretariat of Agriculture, Livestock and Fisheries defines the procedure for determining whether a crop derived from NBTs is a GMO (Gao *et al.*, 2018). Further, the National Commission Advisor in Agricultural Biotechnology (CONABIA) evaluates and regulates NBT‐modified crops submitted by applicants and considers whether these products derived from breeding procedures contain new combination of genetic material. According to the evaluation guidelines from Argentina (Whelan and Lema, 2015), if the modified plant does not contain an adequate entity that qualifies as a novel combination of genetic material and if transgenes were removed from the crop prior to its commercialization, then the applicant is informed that the crop is not subjected to the regulatory framework that is applied to GMOs. However, if transgenes are not removed from the final product or if the product retains a novel combination of genetic material, the plant will be regulated as described in the GMO resolution (Gao *et al.*, 2018). Thus, the regulatory framework of Argentina aspires to assess GMOs as novel products and not by focusing on the procedure used to obtain the GMOs.

## Regulatory perspective in Chile

Chile initiated a working group for different governmental agencies of the Agricultural Ministry to reflect the potential of NBTs. General procedures were published for case‐by‐case evaluations in July of 2017. The Chilean framework has focused on establishing analytical processes to apply the 1523/2001 resolution to products developed using new technologies. According to this process, if these materials fall out of the normative, they are exempted from GM scope and released as conventionally bred plants. The Forestry and Agricultural Protection Division of the Agricultural and Livestock Service (SAG) performs the case‐by‐case analysis of the products obtained from the NBTs (Eriksson *et al.*, 2019).

## Regulatory perspective in New Zealand

Genetically modified organisms in New Zealand (NZ) are regulated by a strict process‐based regulatory framework under the Hazardous Substances and New Organisms (HSNO) Act 1996. HSNO describes GMO very broadly as ‘the organisms in which the genes or genetic material has been altered via in vitro techniques’. Therefore, many techniques that were being used at the time when the Act was approved fell under this broad definition (Fritsche *et al.*, 2018). The HSNO Act, under section 26, describes the procedure for applicants to use when consulting with EPA to determine whether their organism/product is regulated as GM in NZ (Kershen, 2015). However, in April 2015, the EPA stated that the non‐transgenic genome editing mechanism recommended by Scion had similarities to chemical mutagenesis and genetic manipulation. As modifications involving the application of a chemical agent—a protein, do not contain foreign DNA, they are more similar to chemical mutagenesis than genetic manipulation (EPA, 2013; Fritsche *et al.*, 2018). Therefore, in NZ, organisms produced by genome editing should be exempted from the regulatory framework and should not be considered GMOs. Nonetheless, the Sustainability Council of NZ appealed the EPA decision in the High Court. The Court concluded that the list of techniques stated in the HSNO Regulations 1998 is a closed list and any addition to the list of exceptions would be a political judgement, not the administrative decision (Kershen, 2015). Thus, the initial decision of EPA was quashed and the products of all genome editing techniques are now regulated as GM in NZ (Fritsche *et al.*, 2018).

## Regulatory perspective in Japan

In Japan, discussions among the regulatory authorities are continuing. In August of 2018, the Advisory Panel on Genetically Modified Organisms from Japan’s Ministry of Environment held its second meeting to review recommendations made by the expert committee on the handling of genome editing technology. The advisory panel concluded that any living organism with foreign nucleotide(s) remaining in the host genome should be regulated, regardless of whether the foreign nucleotide(s) is detectable. As a result, SDN‐1 edits will fall outside of the scope of the existing regulations on GM plants in Japan (Zannoni, 2019).

## Conclusion and perspectives

In this paper, we have discussed the CRISPR‐Cas system as an instrumental and revolutionary genome editing tool because of its precision, robustness, simplicity and diverse applications. The recent advances in the CRISPR‐Cas system are challenging human values, especially genome editing experiments on human embryos. Using CRISPR‐Cas system, the scientific community must firmly hold to the ethical guidelines established for the safe translation of scientific advances to human health. Here, we discussed ethical and regulatory regimes of various nations for plant genome editing, with an emphasis on genome editing using the CRISPR‐Cas system. Regulatory perspectives vary from nation to nation, such as in the EU and New Zealand gene‐edited products and organisms are assessed on the bases of procedure used to create them. Based on this process‐based approach, genome editing plants are considered GM and are regulated under GMO legislation. In contrast, the United States and most of other countries evaluate genome‐modified products and organisms based on the characteristics of the final product (product‐based approach). Using this approach, if the end product does not contain a foreign entity, such as a transgene, it is not classified as GM and is not regulated under GMO legislation.

We argue that these divergent and strict regulatory frameworks, especially the regulatory framework in the EU, will restrict the application of this highly efficient technology and ultimately will limit the creation of highly profitable crops. To overcome these constraints, we suggest that gene‐edited organisms containing modifications to native genes and lacking foreign transgenes—particularly when foreign transgenes have not been stably integrated into the genome—should be exempted from the restrictive GMO regulatory framework (Fears and Ter Meulen, 2017; Huang *et al.*, 2016; SBA, 2015). In addition, there must be full transparency for disclosing the procedure used to create new crop plants. Since, nucleases are used in the gene editing process to create DSB at the specific target site within the genome, the plants in which repair mechanism leads to a mutation at the target site are selected. In fact, the plants created in this way are on a product‐based approach similar to those created with mutation breeding, which should be considered as conventionally bred plants. In contrast, these products are on a process‐based criterion regulated as GM. However, the objective should be to regulate traits/products and organisms developed rather than to regulate the particular techniques used (EASAC, 2017; Duensing *et al.*, 2018).

We also argue that, although ethical and regulatory concerns for the applications of genome editing technology have already been discussed globally and have established some guidelines, laws and principles to proceed genome editing applications including the use of CRISPR‐Cas system. But based on the current scenario of CRISPR‐Cas applications, these principles and guidelines are not enough for the safe use of this technology particularly in human gene editing. Because the technique is not properly developed and understood yet, and it has technical issues as well. Moreover, in many countries biotechnology policies are outdated and even the policies for genome editing specially CRISPR‐Cas applications for clinical, reproductive and agricultural purposes are poorly discussed. Therefore, more deliberative and global discussion is needed to reconsider and reframe existing ethical guidelines and regulatory policies to develop more scientific and technical worldwide criteria for genome editing with CRISPR‐Cas system. In addition, the nations should strictly bind to international genome editing laws before proceeding any genome editing application with CRISPR‐Cas system in order to promote safety of humankind, other animals and non‐living environment and safe agriculture.

## Conflicts of interest

We confirm that all the authors in our manuscript have no conflicts of interest.

## Author contributions

Debin Zhang, Amjad Hussain and Hakim Manghwar drafted and wrote the manuscript. Kabin Xie, Shengsong Xie, Shuhong Zhao, Shuangxia Jin and Fang Ding revised the manuscript. Robert M. Larkin, Ping Qing and Fang Ding suggested and finally revised the manuscript. Shuangxia Jin and Fang Ding conceived the study.
